# Content Validity of CASA-Q Cough Domains and UCSD-SOBQ for Use in Patients with Idiopathic Pulmonary Fibrosis

**DOI:** 10.5539/gjhs.v5n6p131

**Published:** 2013-09-16

**Authors:** Katharine Gries, Dirk Esser, Ingela Wiklund

**Affiliations:** 1 United BioSource Corporation, Seattle, USA; 2 Boehringer Ingelheim GmbH, Ingelheim am Rhein, Germany; 3 United BioSource Corporation, London, UK

**Keywords:** content validity, idiopathic pulmonary fibrosis, CASA-Q, UCSD-SOBQ, patient-reported outcome

## Abstract

**Purpose:**

The study objective was to assess the content validity of the Cough and Sputum Assessment Questionnaire (CASA-Q) cough domains and the UCSD Shortness of Breath Questionnaire (SOBQ) for use in patients with Idiopathic Pulmonary Fibrosis (IPF).

**Methods:**

Cross-sectional, qualitative study with cognitive interviews in patients with IPF. Study outcomes included relevance, comprehension of item meaning, understanding of the instructions, recall period, response options, and concept saturation.

**Results:**

Interviews were conducted with 18 IPF patients. The mean age was 68.9 years (SD 11.9), 77.8% were male, and 88.9% were Caucasian. The intended meaning of the CASA-Q cough domain items was clearly understood by most of the participants (89–100%). All participants understood the CASA-Q instructions; the correct recall period was reported by 89% of the patients, and the response options were understood by 76%. The intended meaning of the UCSD-SOBQ items was relevant and clearly understood by all participants. Participants understood the instructions (83%) and all patients understood the response options (100%). The reported recall period varied based on the type of activity performed. No concepts were missing, suggesting that saturation was demonstrated for both measures.

**Conclusions:**

This study provides evidence for content validity for the CASA-Q cough domains and the UCSD-SOBQ for patients with IPF. Items of both questionnaires were understood and perceived as relevant to measure the key symptoms of IPF. The results of this study support the use of these instruments in IPF clinical trials as well as further studies of their psychometric properties.

## 1. Introduction

Idiopathic pulmonary fibrosis (IPF) is a chronic, progressive, debilitating fibrotic lung disease, of unknown origin, which is the most common of the idiopathic interstitial pneumonias (Raghu et al., 2011). The clinical course is variable and difficult to predict for the individual patient: while some patients experience rapid disease progression and lung function decline, the condition may remain stable or progress more slowly in others (Tzilas, Koti, Papandrinopoulou, & Tsoukalas, 2009). The most frequent symptoms are shortness of breath and dry cough, but some patients may also suffer from constitutional symptoms like weight loss, anorexia, weakness, or fatigue (American Thoracic Society [ATS], 2000).

Patients with IPF have substantial impairment in health-related quality of life (HRQoL), particularly in the domains of physical health and level of independence, and a number of patient reported outcome (PRO) tools have become available to measure this impairment (Swigris, Kuschner, Jacobs, Wilson, & Gould, 2005). The St. George’s Respiratory Questionnaire (SGRQ), originally developed for use with patients with COPD, has been adapted for assessment in IPF (as SGRQ-I), and more recently, an IPF-specific HRQoL instrument, the ATAQ-IPF (A Tool to Assess QOL in IPF), and an interstitial lung disease (ILD)-specific HRQoL instrument, the King’s Brief Interstitial Lung Disease (K-BILD) have been developed (Patel et al., 2012; Swigris & Fairclough, 2012; J.J. Swigris et al., 2010; Yorke, Jones, & Swigris, 2010). However, these instruments are not a suitable option for the specific assessment of the major symptoms (cough and shortness of breath) in patients with IPF, which would be of advantage as an endpoint in clinical trials. Although each of the instruments mentioned above contains questions on symptoms, their focus is rather on a broad assessment of health status and none of these can be considered to be a symptom tool.

Although there are no fully validated PRO symptom tools for use with patients with IPF, there are several instruments that have been developed to assess cough or shortness of breath in patients with other respiratory diseases. Amongst these, the cough domains of the Cough and Sputum Assessment Questionnaire (CASA-Q) (Crawford et al., 2008; Monz et al., 2010) and the UCSD Shortness-of-Breath Questionnaire (UCSD-SOBQ), (Eakin, Resnikoff, Prewitt, Ries, & Kaplan, 1998; Fabbri et al., 2009) both being comparatively short instruments developed to assess common symptoms of COPD, are of particular interest also for an IPF population. The two cough domains of the CASA-Q allow assessing the level of cough as a symptom in addition to its impact on the patient, in contrast to more generic cough questionnaires such as the Leicester Cough Questionnaire (LCQ) and the Cough Quality of Life Questionnaire (CQLQ) that only explore the impact of cough on health status but do not quantify the level of symptoms as such (Birring et al., 2003; French, Irwin, Fletcher, & Adams, 2002; Lechtzin, Hilliard, & Horton, 2013). The UCSD-SOBQ is also mainly a tool focused on the level of shortness of breath as a symptom; furthermore, there is evidence about its construct validity with IPF patients (Swigris et al., 2010; Swigris & Fairclough, 2012). Therefore the objective of this study was to conduct qualitative research to examine the content validity of the CASA-Q cough domains and UCSD-SOBQ for use in IPF patients and to assess the relevance and comprehensibility of the items of these existing symptom specific questionnaires originally not developed with and for IPF patients.

## 2. Methods

### 2.1 Qualitative Methods

Cognitive interviewing is used to address if the content of a PRO instrument captures the most important aspects of the concept(s) of interest. Additionally, information confirming that respondents understand the questions, response options and recall period are adequate, and understand how to complete the instrument are addressed in cognitive interviewing (Patrick et al., 2011a). These are two of the most important issues emphasized in the FDA PRO Guidance to Industry (Food and Drug Administration, 2009).

The study consisted of one-on-one interviews. Participants completed the CASA-Q cough domains and the UCSD-SOBQ, followed by debriefing questions using a semi-structured interview guide. The interview guide contained questions about the participant’s understanding of the instructions for each instrument, the recall period, the intended meaning and relevance of the items and response options, and general questions about the overall instrument and missing concepts. Institutional review board approval (Ethical and Independent Review; 11005-01D) was obtained prior to the start of the study. All participants provided written informed consent prior to participation.

### 2.2 Participants

Participants were recruited from four clinical sites located in the United States. Participants included needed to be 40 years of age or older, diagnosed with IPF confirmed by HRCT within 5 years from study visit, a forced vital capacity (FVC) greater or equal to 50% predicted, and a carbon monoxide diffusing capacity (DLco) less than 80% but greater or equal to 30% predicted. Participants were excluded if they had a history of alcohol or substance abuse, a recent myocardial infarction, new diagnosis of unstable angina, or had a medical or psychiatric condition that interfered with completing the study visit.

### 2.3 Study Measures

#### 2.3.1 Patient Reported Outcome Instruments

The CASA-Q was developed and validated in study participants with COPD or chronic bronchitis to assess the impact of cough and sputum on patients’ lives. The cough domains of the CASA-Q include 11-items that ask about cough and its impact on daily activities. Participants complete the CASA-Q cough symptom and cough impact domains using a 7-day recall. Response options range from “not at all/never” to “a lot/always” on a five-point scale. Domain scores range from 0 to 100; a higher score is associated with fewer symptoms/less impact due to cough (Crawford et al., 2008). The 9 questions of the CASA-Q sputum domains were not included for this assessment, as cough is usually reported to be dry in IPF patients (ATS, 2000).

The UCSD-SOBQ was originally developed for use in patients with lung disorders and has been applied in pulmonary rehabilitation programs. Additional research supports the validity of the UCSD to assess change in dyspnea over time in patients with IPF (Swigris et al., 2012). The UCSD-SOBQ is a 24-item questionnaire that asks the patients to rate their breathlessness for a given activity for an average day during the past week. The UCSD-SOBQ response options range from “0/not at all” to “5/maximally or unable to do because of breathlessness.” Scores range from 0 to 120; a higher total score is associated with greater severity of shortness of breath (Kupferberg, Kaplan, Slymen, & Ries, 2005).

#### 2.3.2 Sociodemographic and Clinical Characteristics Questionnaire

For descriptive purposes, all participants were asked to complete a sociodemographic and clinical characteristics questionnaire asking about age, gender, ethnic/racial background, employment status, education, smoking status, alcohol status, pulmonary function test, medical conditions, and medications.

### 2.4 Analysis

Descriptive summary statistics were used to characterize the participant sample in terms of sociodemographic and clinical characteristics. Interviews were audio recorded, with the permission of the study participant, and transcribed. A qualitative analysis software program, ATLAS.ti was used as a tool to systematically organize and categorize the qualitative data in textual form (Muhr, 2004). The study team developed a coding dictionary, prior to analysis of the transcripts, to evaluate the content validity for the CASA-Q cough domains and the UCSD-SOBQ (Patrick et al., 2011a, 2011b). The coding dictionary provided a guide in the subjective interpretation of intended meaning of items, instructions, response options, and appropriate use of the recall period.

The qualitative interviews were examined for major themes related to the content validity of the CASA-Q cough domains and the UCSD-SOBQ. The data was analyzed to determine the level of participants’ understanding of the instructions, items, recall period, and response options as well as, the appropriateness of each item for IPF. As part of the content analysis, the level of saturation was assessed, described as no additional data uncovered that demonstrated that any substantially new or previously unrecognized issues or concepts were uncovered. Missing data were excluded from the analysis set.

## 3. Results

### 3.1 Sociodemographic and Clinical Characteristics

The study population included 18 participants with a mean age (SD) of 68.9 (11.9) (range: 43 to 85 years). A majority, 77.8%, were male and Caucasian (n=16, 88.9%) (Table 1). The mean (SD) time since IPF diagnosis was 2.4 (1.6) years. The mean (SD) forced vital capacity (FVC) was 2.9 (0.7) L or 87% (30.7) of the predicted value, and the diffusion capacity (DLco) was 48.7 (15.1) percent predicted.

**Table 1:** Demographic and clinical characteristics of the study population (n=18)

Age; mean (SD)	68.9 (11.9)
Male Gender (n, %)	14 (77.8%)
Race (n, %)	
White	16 (88.9%)
Black or African American	1 (5.6%)
Other	1 (5.6%)
Lung Function FEV_1_; mean L (SD) FVC; mean L (SD)	2.3 (0.6) 2.9 (0.7)
Predicted FEV_1_; % (SD)	89.7 (37.6)
Predicted FVC; % (SD) FEV_1_/FVC; mean (SD)	87.2 (30.7) 0.80 (0.09)
DLco; mean ml/min/mmHg (SD)	11.8 (4.3)
Self-reported overall health	
Very good/Good	10 (55.5%)
Fair/Poor	8 (44.5%)

Scores from the CASA-Q demonstrate the existence of cough symptoms and their impact on patients with IPF. The mean (SD) score for the symptom domain was 46.8 (19.2) and 57.1 (22.3) for the impact domain. These scores were slightly lower (worse) than those observed in patients with COPD or chronic bronchitis (Crawford et al., 2008). The mean (SD) score for the UCSD-SOBQ was 43.1 (23.6). The study population had a slightly lower severity of shortness of breath compared to patients with chronic lung disease, severe emphysema (Giardino et al., 2010), and COPD (Eakin et al., 1998; Kupferberg et al., 2005). Item frequency for the CASA-Q cough domains and the UCSD-SOBQ are included in the online Appendix.

### 3.2 Debriefing Interviews

#### 3.2.1 CASA-Q Cough Domains

**Figure 1:**
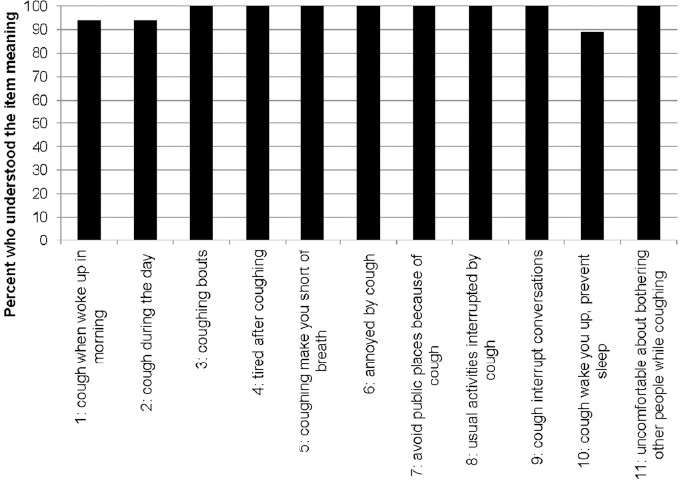
Participants’ ability to understand and explain the meaning of each CASA-Q item (n=18)

The intended meaning of the items was clearly understood by most of the participants (89–100%) (Figure 1). Two participants (11%) reported difficulty in understanding Item 10. One participant indicated that they could not differentiate between “falling asleep” and “falling back to sleep.” The other participant indicated that “waking up,” “preventing you from falling asleep,” and “preventing you back to sleep” were the same concept. For Item 1 and 2, one participant was either not asked or did not reply to the question during the interview, resulting in missing data. The major themes with illustrative quotes are presented in Table 2.

**Table 2:** CASA-Q cough domain themes and participant quotes

Concept	Participant Quote
Morning cough	*Well, I, I seem to um, cough a lot when I get out of bed, and it, it does go away once you’re up moving around and um, you know, you take your medicine and so on. The more you’re moving around, but I have a lot of coughing and choking in the morning, and then it dies down, so, um, it’s the reason I put ‘a lot’. I, um, I seem to have that coughing more in the morning when I get up.*
Daytime cough	*That’s another one that’s sometimes. You can’t um, yeah, I, but um, well, you might go a whole day, you know, it’s like, um, you might do two or three times in a day, you know, um, on an average, or something like that.*
Coughing bouts	*Well, to me, a coughing bout is a serious type of cough where you, you can’t breathe, you have trouble catching your breath, you know, tears are coming down. I don’t have anything like that right now.*
Tired after coughing	*Uh, just regular coughing, no, but if it’s a bout, extremely tired-until I’m exhausted.*
Short of breath	*Well, I think it was often, because once you start the coughing spell you’re, sometimes you’re gasping for breath, and I would say that it’s quite often. I don’t it’s not ‘rarely’ or ‘sometimes’. It’s you when you’re coughing, it just, it doesn’t help you with the shortness of breath.*
Annoyed	*It’s, it, I find it very, very annoying. Not only by myself, but my relatives, my wife. It’s very annoying and I keep apologizing for my coughing*
Avoid public places	*Yeah. More specifically in the past. But today I still am self conscience about coughing. Especially standing in line at the grocery store. Movie theaters, we just did that last week. Or at restaurants especially too. That’s another good one where people don’t want to hear coughing.*
Usual activities interrupted	*Uh, like I said, doing housework. I have to stop in the middle of stuff and settle.*
Interrupt conversations	*Sometimes, uh, when I’m talking on the phone, if I’m talking too long, uh, the experience I’ve had with that is with my daughter. My daughter lives in [name of state] and we talk daily, and if we talk too long I will begin to cough and she’ll tell me to hang up.*
Sleep	*Of course you were asleep but, the cough was bad enough to actually wake you up to where you’re conscious of your coughing. Then to prevent you from falling asleep, it’s sometimes it’s just as you’re about to go to sleep then you start coughing and-and of course it keeps you from falling asleep and falling back to sleep, same thing. ‘Cause if you had-if the cough woke you up then your heart rate’s up and your breathing is-is fast and so it’s difficult to go back to sleep.*
Uncomfortable about bothering others	*My thinking was-is how, uh-oh, which word that I’m-how aware am I of bothering other people. Which I’m very uncomfortable, if I’m bothering other people and interrupting the-their conversation or a movie or church service or, sitting in a waiting room, where it’s quiet.*

**Figure 2:**
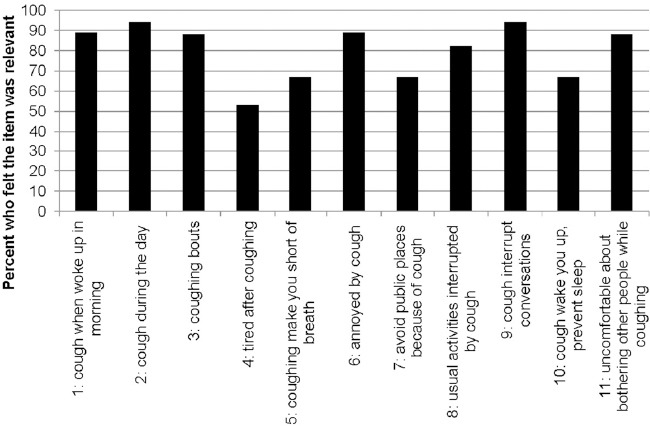
Participants who reported the CASA-Q item was relevant for their IPF experience (n=18)

All the cough items were generally perceived as highly relevant (Figure 2). The individual CASA-Q cough domain items showed that more than half of the population suffered coughing bouts, coughed frequently during the day, were short of breath due to coughing or had to interrupt their activities because of coughing, and found the symptom generally annoying (Table 2). Between 20% and 45% complained about being tired after coughing and also felt that they avoided going places because of coughing.

The CASA-Q instructions were well understood by all participants. The recall period was accurately used by 89% (n=16) of the study participants. A majority of the participants (n=13 of 17, 76%) demonstrated understanding of the response options. The remaining 24% (n=4) demonstrated difficulty in distinguishing specific response options, such as, distinguishing “rarely” from “sometimes,” distinguishing “quite a bit” from “a lot,” distinguishing “somewhat” from “a little,” and “often” from “always.”

#### 3.2.2 UCSD-SOBQ

**Figure 3:**
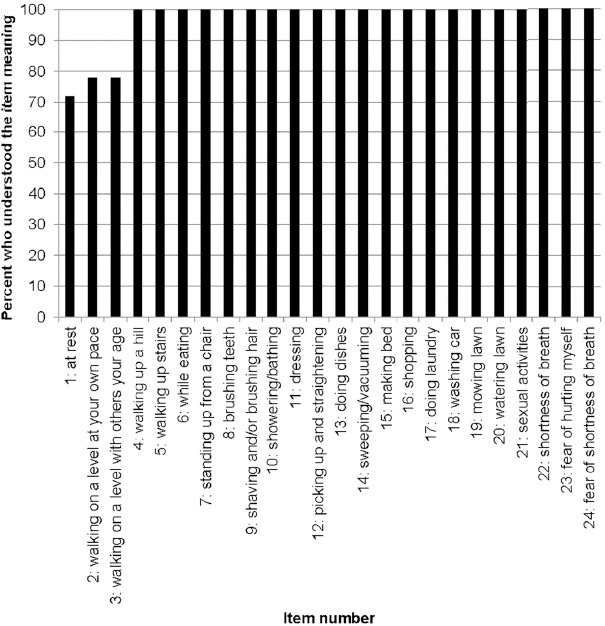
Participants’ ability to understand and explain the meaning of each UCSD-SOBQ item (n=18)

All participants understood the intended meaning of Items 4–24 (Figure 3). For Item 1 (at rest), when probed on the participants’ understanding of the item meaning, two participants (11%) suggested a change to the question to enhance the interpretability in the meaning: omitting the item (n=1), and adding an activity that relates to being at rest, such as watching television (n=1). There was missing data for 5 participants, from either not being asked or no response, for Item 1. For Item 2 (walking on a level at your own pace), when probed on the intended item meaning, 3 participants (16%) did not respond while one participant expressed difficulty in the interpretation of the phrase “on a level.” For Item 3 (walking on a level with others your age), when probed on the intended item meaning, 3 participants (16%) failed to respond and one participant felt that Item 3 and Item 2 were addressing the same concept. The major themes with illustrative quotes are presented in Table 3.

**Table 3:** UCSD-SOBQ themes and participant quotes

Concept	Participant Quote
Walking	*Well, it’s just I know from experience now that I’ve had this for a couple of years, that walking up a hill is going to bother me. If I’m walking up the hill and I’m walking by myself, walking at my own pace, you know, depending on the steepness of the hill I might have to rest halfway up the hill.*
Self-care	*Putting on pantyhose-just dressing period, I just get so winded-I don’t know why. I don’t understand that either-because I really don’t understand why unless I’m just in too big of a hurry-I don’t know.*
	*I think it’s, brushing hair is the-little bit one that I was thinking about more specifically. And it’s just one of those things when you are reaching up and doing something like that you’re doing a little bit of slight physical exertion but it’s again, one of those things that you don’t think about ‘til you’re at that condition.*
Usual activities	*Yeah. Picking up and straightening. Picking up my socks. Yeah, that’s-sometimes that’s difficult. And straightening things up it’s-is typical household chores. And sometimes it’s easy and sometimes it’s difficult. And it depends on how long you’re going to do it. So, it’s your, the time that you’re doing it and how much it affects you.*
	*First of all I usually don’t make the bed, my wife does. If I had to make the bed, I would be thinking the same thing, you know, slinging your arms around, bending over, picking up sheets or pillows or whatever it is-that might affect you some.*
Sexual activities	*The only question that I have is I look at it and think of tiredness and, um, does it affect the sexual activities? Yes, it does. And uh, that’s important too. It’s not, essential but it’s very important to your wellbeing.*
Fear	*The fear of it would be how’s that going to affect-what is this task going to have to do with-is it going to cause me to be shortness-have a shortness of breath. And that would be the only fear. It wouldn’t be-the physical part of it is whether or not I could actually do this task. And I would think that would be the fear.*
	*Yeah. When I was working full time at times I would get out of breath and be in a dangerous situation, uh, on top of a truck or underneath one with a heavy object. And I was afraid that I’d get very dizzy and either fall or pass out and lose the grip on something heavy I was holding. So, the job that I had, uh, and even today I think about passing out if I-or becoming at least dizzy.*

**Figure 4:**
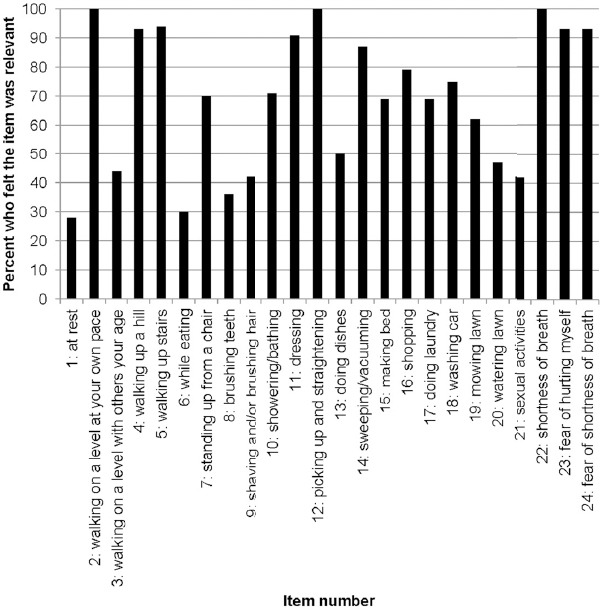
Participants who reported the UCSD-SOBQ item was relevant for their IPF experience (n=18)

The items of the UCSD-SOBQ were relevant to patients, especially questions related to difficulties in performing common day-to-day activities and more strenuous activities (Figure 4). Relevance of items varied, resulting from numerous possible explanations such as the low exertion required for activity, a possible gender role effect, and activities specific to age limitations.

The majority of participants (83%) were able to demonstrate that they understood the UCSD-SOBQ instructions. The recall period drew mixed responses from the participants. About half (46%) reported that they were thinking about an average day over the past week, 15% reported an average day, 15% reported over the past month, and 23% reported over the past couple of years. Participants who used a recall greater than a week avoided the activity or did not engage in the activity because of breathlessness so the participant thought of a longer duration to include the last time the activity was performed. All of the study participants understood the anchor response options (“0: not at all breathless” and “5: maximally breathless”). Five participants (28%) demonstrated difficulty in describing the middle options; the difficult combinations were “1, 2 and 3,” “2 or 3,” and “3 or 4.”

## 4. Discussion

The primary objective of this study was to examine the content validity of the CASA-Q cough domains and the UCSD-SOBQ and assess the relevance of the items in patients with IPF applying the most recent scientific methodology (Patrick et al., 2011a, 2011b). To our knowledge, this is the first study demonstrating evidence of content validity of PRO symptom tools with IPF patients. The eligibility criteria of the study population interviewed was representative of an IPF population included in clinical trials as well as a general IPF population in terms of age, gender, and clinical characteristics supporting the applicability of the tools in the intended sample of IPF patients (Baumgartner, Samet, Stidley, Colby, & Waldron, 1997; Gribbin, Hubbard, & Smith, 2009; Noble et al., 2011; Taskar & Coultas, 2006).

The results of the cognitive debriefing interviews suggest that the items in the CASA-Q cough domains and items in the UCSD-SOBQ were understood and relevant for a majority of the participants. Scores from the CASA-Q cough domains and the UCSD-SOBQ confirmed that cough and shortness of breath are prevalent problems in people with IPF which has severe adverse impacts on several aspects of HRQoL (Martinez et al., 2000). In comparison to COPD patients, the patients with IPF reported less breathlessness. The UCSD-SOBQ was developed with COPD patients participating in a rehabilitation program; these tend to be more active and hence might suffer from more breathlessness than the IPF patients in our study. Generally, respondents had few issues understanding the wording, response options, instructions for the CASA-Q and UCSD-SOBQ instruments. The slightly higher number of respondents quoting a longer recall period for the UCSD-SOBQ appears to be a result of the extrapolation required to answer the hypothetical way the questions are posed (inability to perform a certain activity). There were varying opinions of the appropriateness of items for the UCSD-SOBQ, as expected given the specific nature of the items, diversity in IPF severity and activity level, as well as the comparatively high age of many of the participants. While the study population may have not engaged in these activities because of gender roles or age limitations, it does not imply that they are inappropriate for the IPF population. While the CASA-Q cough domains are specific to cough, the UCSD-SOBQ addresses limitation for a wide range of activities that require a range of exertion.

The patient interviews clearly support the validity and relevance of assessing cough and dyspnea in IPF patients. Both physical and psychological well-being of patients with IPF are significantly impaired compared to the general population particularly in the domains of physical health and level of independence, similar to findings in COPD populations (Ries, 2006; Swigris, Stewart, Gould, & Wilson, 2005). The content of the CASA-Q cough domains and the UCSD-SOBQ was highly relevant as illustrated by the many patients who complained about cough related problems or described difficulties in performing a range of day to day activities or inability to do more strenuous activities thereby supporting previous findings of decrease in physical functioning and increased need for assistance, two important aspects of HRQoL. This study shows that the contents of both the CASA-Q cough domains and the UCSD-SOBQ are valid for IPF and that both measures appear to be suitable for the assessment of the key symptoms of IPF in clinical trials. Data from these trials should be used to gather additional information about their measurement properties, such as construct validity, reliability, and responsiveness, in particular for the CASA-Q cough domains.

Some consideration of the following limitations should be kept in mind. For instance, due to several factors common during an interview there were results that do not include the entire sample. Some of these variables include: running out of time, the participant did not understand a previous concept so subsequent questions were not asked, or the probe could have been skipped for reasons deemed appropriate by the interviewer. Particularly in a population with IPF, the age and severity of illness may have attributed to the participant being unable to respond to the question, especially if their disease state prevents them from participating in the activity in question. Also important to note is there were varying degrees of IPF severity in the study population. A heterogeneous disease population is ideal in eliciting varying perspectives and captures a greater reality of the disease state, but risks the chance of including participants who feel very limited and participants with only minor limitations. The study included a small sample of participants recruited from the United States. Extrapolation to patients from other countries and/or cultural background has to be done with care and in accordance with international standards (Wild et al., 2009). The external validity of the results would be enhanced with an increase in sample size and greater diversity in the gender distribution and race/ethnicity of the sample.

The current study assesses the content validity of the CASA-Q cough domains and the UCSD-SOBQ for IPF. While these PRO instruments address the most prominent symptoms of IPF, they do not include some of the other major symptoms such as chest pain, weight loss, weakness, and fatigue. Additional research would be beneficial in assessing the impact of these symptoms on patients’ lives and the validity of these symptoms in a PRO instrument for IPF.

## 5. Conclusion

The content validity of the CASA-Q cough domains and the UCSD-SOBQ in IPF patients is well established in the cognitive interviews. The CASA-Q cough domain and UCSD-SOBQ were relevant for the measurement of the key symptoms of idiopathic pulmonary fibrosis and their impact on patients, and the content of the instructions, items, and response scales were clearly understood by nearly all participants. The results of this study provide evidence supporting use of the CASA-Q cough domain and UCSD-SOBQ in clinical trials for patients with IPF (Fabbri et al., 2009; Meek & Lareau, 2003; Verrill, Barton, Beasley, & Lippard, 2005).
